# 5-Bromo-2-(thio­phen-2-yl)-1-(thio­phen-2-ylmeth­yl)-1*H*-benzimidazole

**DOI:** 10.1107/S1600536812042146

**Published:** 2012-10-13

**Authors:** David K. Geiger, Matthew R. Destefano

**Affiliations:** a Department of Chemistry, State University of New York-College at Geneseo, 1 College Circle, Geneseo, NY 14454, USA

## Abstract

There are two independent mol­ecules in the asymmetric unit of the title compound, C_16_H_11_BrN_2_S_2_. In the crystal, weak C—H⋯N hydrogen bonds and C—H⋯thio­phene ring inter­actions link the mol­ecules into chains along [100]. The structure exhibits disorder of the 2-thio­phen-2-yl substituent of one of the symmetry-unique mol­ecules with a major:minor component ratio of 0.914 (3):0.086 (3).

## Related literature

For the characterization of 2-(thio­phen-2-yl)-1-(thio­phen-2-ylmeth­yl)-1*H*-benzimidazole, see: Geiger *et al.* (2012 ▶). For examples of pharmacological uses of benzimidazoles, see: López-Rodríguez *et al.* (1999 ▶); Varala *et al.* (2007 ▶); Horton *et al.* (2003 ▶). For the synthesis of substituted benzimidazoles, see: Grimmett (1997 ▶).
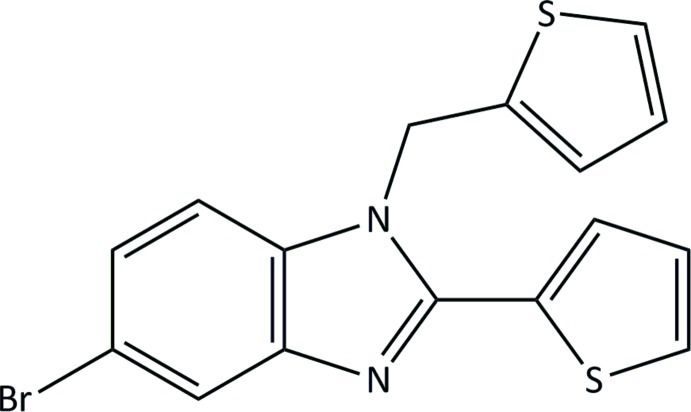


## Experimental

### 

#### Crystal data

C_16_H_11_BrN_2_S_2_
*M* *_r_* = 375.30
Monoclinic, 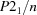

*a* = 12.6753 (17) Å
*b* = 10.5413 (11) Å
*c* = 23.581 (3) Å
β = 100.878 (4)°
*V* = 3094.1 (6) Å^3^
*Z* = 8
Mo *K*α radiation
μ = 2.92 mm^−1^
*T* = 200 K
0.60 × 0.20 × 0.10 mm

#### Data collection

Bruker SMART X2S benchtop diffractometer
Absorption correction: multi-scan (*SADABS*; Sheldrick, 2008*a* ▶) *T*_min_ = 0.46, *T*_max_ = 0.76
19813 measured reflections
5581 independent reflections
4191 reflections with *I* > 2σ(*I*)
*R*_int_ = 0.059

#### Refinement

*R*[*F* ^2^ > 2σ(*F* ^2^)] = 0.038
*wR*(*F* ^2^) = 0.091
*S* = 1.02
5581 reflections
392 parameters
91 restraints
H-atom parameters constrained
Δρ_max_ = 0.65 e Å^−3^
Δρ_min_ = −0.80 e Å^−3^

### 

Data collection: *APEX2* (Bruker, 2010 ▶); cell refinement: *SAINT* (Bruker, 2009 ▶); data reduction: *SAINT*; program(s) used to solve structure: *SHELXS97* (Sheldrick, 2008*b* ▶); program(s) used to refine structure: *SHELXL97* (Sheldrick, 2008*b* ▶); molecular graphics: *XSHELL* (Bruker, 2004 ▶) and *Mercury* (Macrae *et al.*, 2008 ▶); software used to prepare material for publication: *publCIF* (Westrip, 2010 ▶).

## Supplementary Material

Crystal structure: contains datablock(s) global, I. DOI: 10.1107/S1600536812042146/lr2085sup1.cif

Structure factors: contains datablock(s) I. DOI: 10.1107/S1600536812042146/lr2085Isup2.hkl

Supplementary material file. DOI: 10.1107/S1600536812042146/lr2085Isup3.mol

Supplementary material file. DOI: 10.1107/S1600536812042146/lr2085Isup4.cml

Additional supplementary materials:  crystallographic information; 3D view; checkCIF report

## Figures and Tables

**Table 1:** Hydrogen-bond geometry (Å, °) *Cg*2 and *Cg*4 are the centroids of the S2,C13–C16 and S4,C29–C32 rings, respectively.

*D*—H⋯*A*	*D*—H	H⋯*A*	*D*⋯*A*	*D*—H⋯*A*
C12^i^—H12*B* ^i^⋯N4	0.99	2.57	3.454 (4)	149
C22—H22⋯N2	0.95	2.61	3.504 (4)	158
C28—H28*A*⋯N2	0.99	2.61	3.522 (4)	152
C6^i^—H6^i^⋯N4	0.95	2.65	3.547 (4)	157
C3—H3⋯*Cg*4	0.95	2.68	3.578 (4)	158
C19—H19⋯*Cg*2^i^	0.95	2.62	3.512 (4)	157

Symmetry code: (i) 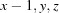.

**Fig. 1.**
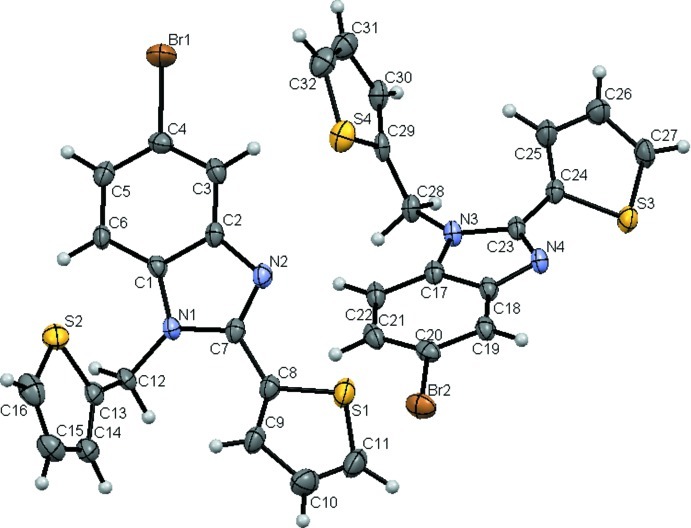
Perspective view of the title compound showing both molecules in the asymmetric unit. Displacement ellipsoids of the nonhydrogen atoms are drawn a at the 50% probability level. Only the major component of the disordered thiophene is shown.

**Fig. 2.**
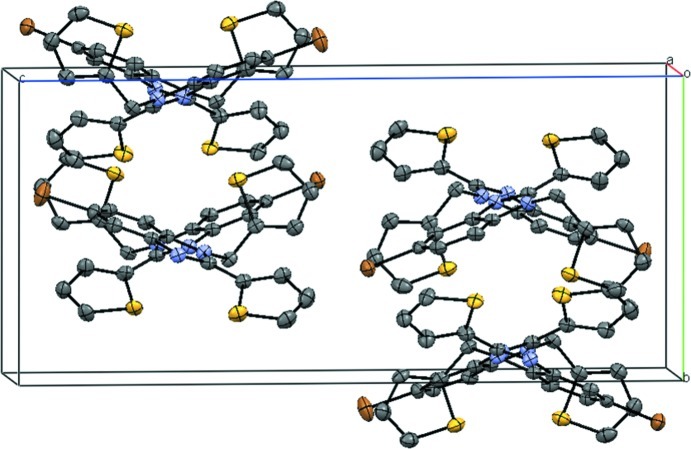
The unit cell of the title compound viewed down the a axis. Hydrogen atoms have been omitted for clarity. Only the major component of the disordered thiophene substituent is shown.

**Fig. 3.**
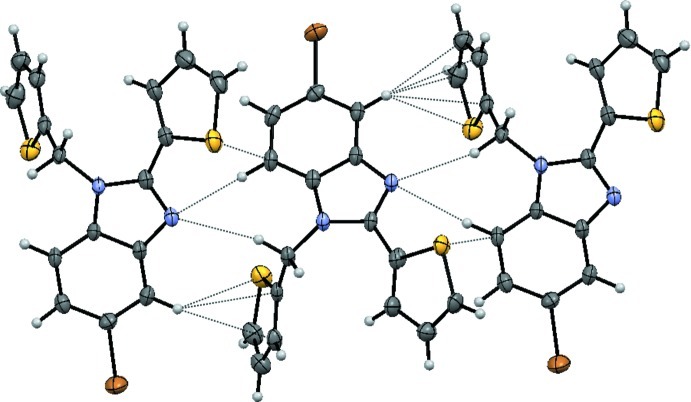
Perspective drawing showing the close intermolecular contacts forming chains parallel to the a axis. Only the major component of the disordered thiophene substituent is shown.

## supplementary crystallographic information

### Comment

Benzimidazole derivatives have a myriad of pharmacological uses, including as inhibitors of serotonin activated neurotransmission (López-Rodríguez *et al.*, 1999) and antiviral agents (Varala *et al.*, 2007). They are also in antiarrhythmic, antihistamine, antiulcer, anticancer, fungicidal, and anthelmintical drugs (Horton *et al.*, 2003).

Numerous methods are available for the synthesis of substituted benzimidazoles (Grimmett, 1997). Our efforts have focused on the preparation of benzimidazole analogues which have substituents capable of binding metals. Toward that end, we have prepared the title compound from a reaction of 1,2-diamino-4-bromobenzene with 2-thiophenecarboxaldehyde. The 5-bromo and 6-bromo substituted benzimidazoles are formed in an approximately 3:2 ratio based on ^1^H NMR spectral data. However, only the 5-bromo isomer forms single crystals under the crystallization conditions employed.

Figure 1 shows a perspective view of the two molecules in the asymmetric unit with the atom-labeling scheme. The molecules exhibit the expected planar benzimidazole moieties with maximum deviations of 0.030 (3) Å (C5) and 0.026 (2) Å (C21) in molecules 1 and 2, respectively. The thiophene rings display maximum deviations from planarity of 0.0004 (23) Å (C10 and C11), 0.005 (2) Å (C16), 0.005 (4) Å (C24), and 0.007 (2) Å (C29).

Figure 2 shows the unit cell as viewed down the *a* axis. Chains of molecules are held together *via* weak C—H···N and *C*—*H*···thiophene ring interactions. The motif is shown in Figure 3. The H19···*Cg*2 and H3···*Cg*4, where *Cg*n refers to the centroid of the thiophene ring containing the sulfur labeled Sn, are 2.62 Å and 2.68 Å, respectively. H19 is 2.618 (3) Å from the thiophene mean plane and H3 is 2.673 (3) Å from the thiophene mean plane.

### Experimental

An approximately equimolar mixture of the 5-bromo and 6-bromo derivatives of the 1,2-disubstituted benzimidazole was prepared by reaction of 500 mg 1,2-diamino-4-bromobenzenene and 0.50 ml 2-thiophenecarboxaldehyde in refluxing dichloromethane (8 ml) in the presence of a catalytic amount of aluminium trichloride for eight hours. After removal of insoluble inorganic material, the solvent was removed by rotary evaporation leaving a brown, tarry substance. The mixture was subjected to column chromatography on silica gel using a 1:4 ethylacetate:hexanes eluent. A light yellow fraction was collected. Based on the presence of two CH_2_ resonances in the ^1^H NMR spectrum, the 5-Br and 6-Br isomers were present in a 3:2 ratio.

Slow evaporation of a 1:4 ethylacetate:hexanes solution at 40°C yielded single crystals of the title compound suitable for X-ray diffraction. A ^1^H NMR spectrum of a solution of single crystals showed that only the 5-Br isomer was present. ^1^H NMR spectrum (CDCl_3_, 400 MHz, p.p.m.): 7.93 (1*H*, s), 7.54 (1*H*, m), 7.48 (1*H*, m), 7.36 (1*H*, m), 7.23 (2*H*, m), 7.15 (1*H*, m), 6.85 (1*H*, bs), 5.68 (2*H*, s).

### Refinement

The H atoms were refined using a riding model with a C—H distance of 0.99 Å for the methylene carbon atoms and 0.95 Å for the phenyl and thiophene carbon atoms. The H atom thermal parameters were set using the approximation *U*_iso_ = 1.2*U*_eq_(C).

During the later stages of refinement, the thiophene ring containing S3 was found to be rotationally disordered. The disorder was resolved using the metrics of the major component to establish coordinates of the minor component. The major:minor site occupancies refined to 0.914 (3):0.086 (3).

### Crystal data

**Table:**

C_16_H_11_BrN_2_S_2_	*F*(000) = 1504
*M**_r_* = 375.30	*D*_x_ = 1.611 Mg m^−^^3^
Monoclinic, *P*2_1_/*n*	Mo *K*α radiation, λ = 0.71073 Å
*a* = 12.6753 (17) Å	Cell parameters from 6104 reflections
*b* = 10.5413 (11) Å	θ = 2.5–24.4°
*c* = 23.581 (3) Å	µ = 2.92 mm^−^^1^
β = 100.878 (4)°	*T* = 200 K
*V* = 3094.1 (6) Å^3^	Plate, colourless
*Z* = 8	0.60 × 0.20 × 0.10 mm

### Data collection

**Table:**

Bruker SMART X2S benchtop diffractometer	5581 independent reflections
Radiation source: XOS X-beam microfocus source	4191 reflections with *I* > 2σ(*I*)
Doubly curved silicon crystal monochromator	*R*_int_ = 0.059
Detector resolution: 8.3330 pixels mm^-1^	θ_max_ = 25.4°, θ_min_ = 2.1°
ω scans	*h* = −15→15
Absorption correction: multi-scan (*SADABS*; Sheldrick, 2008*a*)	*k* = −12→12
*T*_min_ = 0.46, *T*_max_ = 0.76	*l* = −28→24
19813 measured reflections	

### Refinement

**Table:**

Refinement on *F*^2^	Primary atom site location: structure-invariant direct methods
Least-squares matrix: full	Secondary atom site location: difference Fourier map
*R*[*F*^2^ > 2σ(*F*^2^)] = 0.038	Hydrogen site location: inferred from neighbouring sites
*wR*(*F*^2^) = 0.091	H-atom parameters constrained
*S* = 1.02	*w* = 1/[σ^2^(*F*_o_^2^) + (0.036*P*)^2^ + 0.884*P*] where *P* = (*F*_o_^2^ + 2*F*_c_^2^)/3
5581 reflections	(Δ/σ)_max_ = 0.001
392 parameters	Δρ_max_ = 0.65 e Å^−^^3^
91 restraints	Δρ_min_ = −0.80 e Å^−^^3^

### Special details

**Table:**

Geometry. All e.s.d.'s (except the e.s.d. in the dihedral angle between two l.s. planes) are estimated using the full covariance matrix. The cell e.s.d.'s are taken into account individually in the estimation of e.s.d.'s in distances, angles and torsion angles; correlations between e.s.d.'s in cell parameters are only used when they are defined by crystal symmetry. An approximate (isotropic) treatment of cell e.s.d.'s is used for estimating e.s.d.'s involving l.s. planes.
Refinement. Refinement of *F*^2^ against ALL reflections. The weighted *R*-factor *wR* and goodness of fit *S* are based on *F*^2^, conventional *R*-factors *R* are based on *F*, with *F* set to zero for negative *F*^2^. The threshold expression of *F*^2^ > σ(*F*^2^) is used only for calculating *R*-factors(gt) *etc*. and is not relevant to the choice of reflections for refinement. *R*-factors based on *F*^2^ are statistically about twice as large as those based on *F*, and *R*- factors based on ALL data will be even larger.

### Fractional atomic coordinates and isotropic or equivalent isotropic displacement parameters (Å^2^)

**Table:**

	*x*	*y*	*z*	*U*_iso_*/*U*_eq_	Occ. (<1)
Br1	0.75134 (3)	1.11709 (4)	0.458735 (16)	0.05101 (14)	
Br2	0.11272 (3)	1.13960 (3)	0.03496 (14)	0.03685 (12)	
S1	0.41783 (7)	0.73442 (9)	0.17228 (4)	0.0370 (2)	
S2	0.81366 (8)	1.15655 (8)	0.16253 (4)	0.0354 (2)	
S3	−0.03440 (10)	0.73244 (14)	0.31444 (5)	0.0301 (3)	0.914 (3)
C24	0.0711 (5)	0.8382 (6)	0.32690 (16)	0.0225 (9)	0.914 (3)
C25	0.1030 (6)	0.8601 (8)	0.3841 (3)	0.0350 (16)	0.914 (3)
H25	0.1597	0.9164	0.3994	0.042*	0.914 (3)
C26	0.0435 (4)	0.7907 (4)	0.41889 (18)	0.0348 (12)	0.914 (3)
H26	0.0558	0.795	0.4598	0.042*	0.914 (3)
C27	−0.0325 (4)	0.7179 (4)	0.38673 (17)	0.0331 (11)	0.914 (3)
H27	−0.0798	0.6643	0.4026	0.04*	0.914 (3)
S300	0.118 (2)	0.872 (3)	0.3950 (10)	0.0301 (3)	0.086 (3)
C240	0.062 (5)	0.846 (7)	0.3243 (10)	0.0225 (9)	0.086 (3)
C250	−0.013 (4)	0.753 (6)	0.3166 (15)	0.0350 (16)	0.086 (3)
H250	−0.0504	0.7253	0.2801	0.042*	0.086 (3)
C260	−0.028 (4)	0.702 (5)	0.3704 (17)	0.0348 (12)	0.086 (3)
H260	−0.08	0.6398	0.3741	0.042*	0.086 (3)
C270	0.041 (5)	0.753 (5)	0.4156 (15)	0.0331 (11)	0.086 (3)
H270	0.0454	0.7255	0.4544	0.04*	0.086 (3)
S4	0.36606 (8)	1.15559 (8)	0.33291 (4)	0.0359 (2)	
C29	0.3469 (2)	1.0042 (3)	0.35642 (13)	0.0243 (7)	
C30	0.37895 (19)	0.9943 (2)	0.41524 (11)	0.0290 (8)	
H30	0.3759	0.9179	0.4363	0.035*	
C31	0.41740 (19)	1.1124 (2)	0.44115 (11)	0.0372 (9)	
H31	0.4422	1.1238	0.4814	0.045*	
C32	0.4144 (3)	1.2058 (3)	0.40164 (15)	0.0383 (9)	
H32	0.4367	1.2905	0.4111	0.046*	
N1	0.6914 (2)	0.9247 (2)	0.21670 (11)	0.0237 (6)	
N2	0.5457 (2)	0.9232 (3)	0.25919 (11)	0.0259 (6)	
N3	0.2090 (2)	0.9262 (2)	0.27375 (11)	0.0232 (6)	
N4	0.0354 (2)	0.9291 (2)	0.22968 (11)	0.0233 (6)	
C1	0.7240 (2)	0.9759 (3)	0.27135 (13)	0.0222 (7)	
C2	0.6323 (2)	0.9745 (3)	0.29708 (13)	0.0225 (7)	
C3	0.6388 (3)	1.0186 (3)	0.35289 (14)	0.0272 (8)	
H3	0.5777	1.0208	0.3708	0.033*	
C4	0.7381 (3)	1.0590 (3)	0.38108 (13)	0.0279 (8)	
C5	0.8299 (3)	1.0590 (3)	0.35640 (14)	0.0275 (8)	
H5	0.8967	1.0864	0.3783	0.033*	
C6	0.8232 (2)	1.0189 (3)	0.30016 (14)	0.0252 (7)	
H6	0.8839	1.0208	0.2819	0.03*	
C7	0.5840 (3)	0.8946 (3)	0.21240 (14)	0.0241 (7)	
C8	0.5183 (2)	0.8366 (3)	0.16143 (14)	0.0266 (7)	
C9	0.5175 (3)	0.8543 (3)	0.10299 (15)	0.0317 (8)	
H9	0.5664	0.9071	0.088	0.038*	
C10	0.4344 (3)	0.7835 (4)	0.06878 (16)	0.0420 (10)	
H10	0.4213	0.7838	0.0278	0.05*	
C11	0.3754 (3)	0.7156 (3)	0.09998 (16)	0.0399 (9)	
H11	0.3168	0.6631	0.0834	0.048*	
C12	0.7635 (3)	0.8988 (3)	0.17647 (13)	0.0252 (7)	
H12A	0.7361	0.824	0.1528	0.03*	
H12B	0.8352	0.8766	0.199	0.03*	
C13	0.7760 (2)	1.0068 (3)	0.13666 (14)	0.0260 (8)	
C14	0.7663 (3)	1.0014 (3)	0.07791 (14)	0.0310 (8)	
H14	0.7466	0.9271	0.0557	0.037*	
C15	0.7891 (3)	1.1198 (3)	0.05394 (16)	0.0369 (9)	
H15	0.7866	1.1329	0.0139	0.044*	
C16	0.8147 (3)	1.2113 (4)	0.09412 (17)	0.0408 (10)	
H16	0.8314	1.2962	0.0855	0.049*	
C17	0.2065 (2)	0.9796 (3)	0.21996 (13)	0.0228 (7)	
C18	0.0977 (2)	0.9815 (3)	0.19334 (13)	0.0230 (7)	
C19	0.0678 (3)	1.0299 (3)	0.13786 (14)	0.0251 (7)	
H19	−0.0053	1.0341	0.1191	0.03*	
C20	0.1492 (3)	1.0714 (3)	0.11131 (13)	0.0262 (7)	
C21	0.2576 (3)	1.0675 (3)	0.13697 (14)	0.0283 (8)	
H21	0.3104	1.0956	0.1161	0.034*	
C22	0.2882 (3)	1.0226 (3)	0.19272 (14)	0.0273 (8)	
H22	0.3614	1.0211	0.2115	0.033*	
C23	0.1038 (2)	0.8981 (3)	0.27693 (14)	0.0226 (7)	
C28	0.3078 (2)	0.8977 (3)	0.31505 (13)	0.0244 (7)	
H28A	0.365	0.8763	0.2933	0.029*	
H28B	0.2952	0.8218	0.3376	0.029*	

### Atomic displacement parameters (Å^2^)

**Table:**

	*U*^11^	*U*^22^	*U*^33^	*U*^12^	*U*^13^	*U*^23^
Br1	0.0473 (3)	0.0758 (3)	0.0317 (2)	−0.0111 (2)	0.01178 (18)	−0.0179 (2)
Br2	0.0475 (2)	0.0365 (2)	0.02852 (19)	0.00007 (17)	0.01243 (17)	0.00434 (16)
S1	0.0262 (5)	0.0372 (6)	0.0474 (6)	−0.0087 (4)	0.0061 (4)	−0.0036 (4)
S2	0.0361 (5)	0.0294 (5)	0.0426 (5)	−0.0005 (4)	0.0121 (4)	−0.0066 (4)
S3	0.0251 (7)	0.0301 (8)	0.0354 (6)	−0.0096 (4)	0.0067 (4)	0.0013 (4)
C24	0.016 (2)	0.021 (2)	0.0304 (17)	−0.0001 (16)	0.0044 (14)	0.0012 (14)
C25	0.027 (4)	0.044 (4)	0.035 (3)	−0.012 (2)	0.008 (3)	0.000 (3)
C26	0.035 (2)	0.040 (3)	0.030 (2)	−0.009 (2)	0.0067 (17)	0.0000 (19)
C27	0.027 (2)	0.039 (3)	0.035 (2)	−0.0082 (19)	0.011 (2)	0.007 (2)
S300	0.0251 (7)	0.0301 (8)	0.0354 (6)	−0.0096 (4)	0.0067 (4)	0.0013 (4)
C240	0.016 (2)	0.021 (2)	0.0304 (17)	−0.0001 (16)	0.0044 (14)	0.0012 (14)
C250	0.027 (4)	0.044 (4)	0.035 (3)	−0.012 (2)	0.008 (3)	0.000 (3)
C260	0.035 (2)	0.040 (3)	0.030 (2)	−0.009 (2)	0.0067 (17)	0.0000 (19)
C270	0.027 (2)	0.039 (3)	0.035 (2)	−0.0082 (19)	0.011 (2)	0.007 (2)
S4	0.0356 (5)	0.0281 (5)	0.0425 (5)	−0.0021 (4)	0.0030 (4)	0.0085 (4)
C29	0.0116 (16)	0.0272 (19)	0.0346 (18)	0.0006 (13)	0.0053 (14)	0.0074 (15)
C30	0.0213 (18)	0.027 (2)	0.039 (2)	−0.0034 (15)	0.0060 (15)	0.0092 (16)
C31	0.031 (2)	0.039 (2)	0.038 (2)	0.0003 (17)	−0.0014 (17)	−0.0018 (18)
C32	0.036 (2)	0.030 (2)	0.046 (2)	−0.0029 (17)	0.0019 (18)	−0.0006 (18)
N1	0.0152 (14)	0.0286 (15)	0.0279 (14)	−0.0007 (12)	0.0058 (11)	−0.0034 (12)
N2	0.0166 (15)	0.0289 (16)	0.0322 (15)	0.0000 (12)	0.0050 (12)	−0.0014 (12)
N3	0.0155 (14)	0.0252 (15)	0.0297 (14)	−0.0007 (11)	0.0061 (11)	0.0038 (12)
N4	0.0148 (14)	0.0265 (15)	0.0292 (14)	0.0007 (11)	0.0061 (12)	0.0019 (12)
C1	0.0171 (16)	0.0238 (18)	0.0266 (17)	0.0030 (14)	0.0067 (14)	−0.0006 (14)
C2	0.0165 (17)	0.0208 (18)	0.0302 (17)	0.0008 (14)	0.0045 (14)	0.0024 (14)
C3	0.0212 (18)	0.0292 (19)	0.0333 (18)	0.0021 (14)	0.0107 (15)	0.0007 (15)
C4	0.030 (2)	0.029 (2)	0.0252 (17)	0.0014 (15)	0.0072 (15)	−0.0043 (15)
C5	0.0208 (18)	0.0253 (19)	0.0352 (19)	−0.0024 (14)	0.0020 (15)	−0.0021 (15)
C6	0.0151 (16)	0.0267 (19)	0.0348 (18)	−0.0023 (14)	0.0074 (14)	−0.0028 (15)
C7	0.0183 (17)	0.0214 (18)	0.0325 (18)	0.0005 (14)	0.0044 (14)	0.0006 (14)
C8	0.0178 (17)	0.0275 (19)	0.0335 (18)	0.0000 (14)	0.0026 (14)	−0.0014 (15)
C9	0.0187 (18)	0.034 (2)	0.040 (2)	−0.0031 (15)	−0.0003 (15)	−0.0033 (16)
C10	0.034 (2)	0.053 (3)	0.037 (2)	0.0028 (19)	−0.0003 (18)	−0.0079 (19)
C11	0.025 (2)	0.042 (2)	0.050 (2)	−0.0056 (17)	−0.0014 (17)	−0.0116 (19)
C12	0.0171 (17)	0.0298 (19)	0.0306 (18)	0.0018 (14)	0.0093 (14)	−0.0056 (15)
C13	0.0165 (17)	0.0286 (19)	0.0343 (19)	0.0028 (14)	0.0085 (15)	−0.0044 (15)
C14	0.0251 (19)	0.033 (2)	0.0362 (19)	−0.0008 (15)	0.0101 (15)	−0.0055 (16)
C15	0.034 (2)	0.044 (2)	0.034 (2)	0.0089 (17)	0.0108 (17)	0.0057 (18)
C16	0.039 (2)	0.032 (2)	0.056 (2)	0.0057 (18)	0.0203 (19)	0.007 (2)
C17	0.0191 (17)	0.0190 (17)	0.0311 (17)	−0.0001 (13)	0.0067 (14)	−0.0001 (14)
C18	0.0173 (17)	0.0217 (18)	0.0314 (18)	0.0012 (14)	0.0081 (14)	−0.0014 (14)
C19	0.0192 (17)	0.0238 (18)	0.0338 (18)	0.0019 (14)	0.0090 (14)	−0.0020 (15)
C20	0.032 (2)	0.0222 (18)	0.0269 (17)	0.0025 (15)	0.0111 (15)	−0.0013 (14)
C21	0.0270 (19)	0.0242 (19)	0.0378 (19)	−0.0022 (15)	0.0167 (16)	0.0000 (16)
C22	0.0184 (17)	0.0248 (19)	0.0404 (19)	−0.0018 (14)	0.0099 (15)	0.0005 (16)
C23	0.0169 (16)	0.0198 (18)	0.0325 (18)	−0.0020 (13)	0.0085 (14)	−0.0005 (14)
C28	0.0171 (17)	0.0234 (18)	0.0327 (18)	0.0005 (14)	0.0047 (14)	0.0066 (14)

### Geometric parameters (Å, º)

**Table:**

Br1—C4	1.908 (3)	N3—C28	1.465 (4)
Br2—C20	1.912 (3)	N4—C23	1.317 (4)
S1—C11	1.700 (4)	N4—C18	1.384 (4)
S1—C8	1.723 (3)	C1—C6	1.387 (4)
S2—C16	1.716 (4)	C1—C2	1.409 (4)
S2—C13	1.727 (3)	C2—C3	1.384 (4)
S3—C27	1.707 (4)	C3—C4	1.376 (4)
S3—C24	1.723 (4)	C3—H3	0.95
C24—C25	1.352 (6)	C4—C5	1.396 (4)
C24—C23	1.464 (4)	C5—C6	1.379 (4)
C25—C26	1.417 (7)	C5—H5	0.95
C25—H25	0.95	C6—H6	0.95
C26—C27	1.348 (5)	C7—C8	1.461 (4)
C26—H26	0.95	C8—C9	1.389 (5)
C27—H27	0.95	C9—C10	1.412 (5)
S300—C240	1.707 (17)	C9—H9	0.95
S300—C270	1.721 (17)	C10—C11	1.349 (5)
C240—C250	1.353 (17)	C10—H10	0.95
C240—C23	1.430 (17)	C11—H11	0.95
C250—C260	1.422 (17)	C12—C13	1.503 (4)
C250—H250	0.95	C12—H12A	0.99
C260—C270	1.349 (16)	C12—H12B	0.99
C260—H260	0.95	C13—C14	1.369 (4)
C270—H270	0.95	C14—C15	1.422 (5)
S4—C32	1.705 (4)	C14—H14	0.95
S4—C29	1.722 (3)	C15—C16	1.348 (5)
C29—C30	1.374 (4)	C15—H15	0.95
C29—C28	1.509 (4)	C16—H16	0.95
C30—C31	1.4313	C17—C22	1.393 (4)
C30—H30	0.95	C17—C18	1.404 (4)
C31—C32	1.351 (4)	C18—C19	1.388 (4)
C31—H31	0.95	C19—C20	1.375 (4)
C32—H32	0.95	C19—H19	0.95
N1—C7	1.383 (4)	C20—C21	1.393 (4)
N1—C1	1.386 (4)	C21—C22	1.382 (4)
N1—C12	1.461 (4)	C21—H21	0.95
N2—C7	1.321 (4)	C22—H22	0.95
N2—C2	1.387 (4)	C28—H28A	0.99
N3—C23	1.383 (4)	C28—H28B	0.99
N3—C17	1.383 (4)		
			
C11—S1—C8	91.52 (17)	C5—C6—H6	121.4
C16—S2—C13	91.50 (17)	C1—C6—H6	121.4
C27—S3—C24	91.35 (18)	N2—C7—N1	113.4 (3)
C25—C24—C23	130.8 (5)	N2—C7—C8	122.7 (3)
C25—C24—S3	110.9 (4)	N1—C7—C8	123.8 (3)
C23—C24—S3	118.0 (3)	C9—C8—C7	131.0 (3)
C24—C25—C26	113.5 (5)	C9—C8—S1	111.3 (2)
C24—C25—H25	123.3	C7—C8—S1	117.5 (2)
C26—C25—H25	123.3	C8—C9—C10	111.2 (3)
C27—C26—C25	111.7 (4)	C8—C9—H9	124.4
C27—C26—H26	124.2	C10—C9—H9	124.4
C25—C26—H26	124.2	C11—C10—C9	113.5 (3)
C26—C27—S3	112.6 (3)	C11—C10—H10	123.2
C26—C27—H27	123.7	C9—C10—H10	123.2
S3—C27—H27	123.7	C10—C11—S1	112.5 (3)
C240—S300—C270	90.3 (11)	C10—C11—H11	123.8
C250—C240—C23	122 (3)	S1—C11—H11	123.8
C250—C240—S300	113.4 (14)	N1—C12—C13	114.7 (3)
C23—C240—S300	124 (2)	N1—C12—H12A	108.6
C240—C250—C260	111.3 (17)	C13—C12—H12A	108.6
C240—C250—H250	124.3	N1—C12—H12B	108.6
C260—C250—H250	124.3	C13—C12—H12B	108.6
C270—C260—C250	112.5 (18)	H12A—C12—H12B	107.6
C270—C260—H260	123.8	C14—C13—C12	127.0 (3)
C250—C260—H260	123.8	C14—C13—S2	111.2 (3)
C260—C270—S300	112.3 (16)	C12—C13—S2	121.8 (2)
C260—C270—H270	123.8	C13—C14—C15	112.3 (3)
S300—C270—H270	123.8	C13—C14—H14	123.8
C32—S4—C29	91.66 (16)	C15—C14—H14	123.8
C30—C29—C28	126.5 (3)	C16—C15—C14	112.9 (3)
C30—C29—S4	111.3 (2)	C16—C15—H15	123.6
C28—C29—S4	122.1 (2)	C14—C15—H15	123.6
C29—C30—C31	112.13 (16)	C15—C16—S2	112.1 (3)
C29—C30—H30	123.9	C15—C16—H16	123.9
C31—C30—H30	123.9	S2—C16—H16	123.9
C32—C31—C30	112.13 (19)	N3—C17—C22	131.7 (3)
C32—C31—H31	123.9	N3—C17—C18	105.5 (3)
C30—C31—H31	123.9	C22—C17—C18	122.9 (3)
C31—C32—S4	112.8 (3)	N4—C18—C19	130.1 (3)
C31—C32—H32	123.6	N4—C18—C17	110.1 (3)
S4—C32—H32	123.6	C19—C18—C17	119.7 (3)
C7—N1—C1	105.9 (2)	C20—C19—C18	116.9 (3)
C7—N1—C12	129.5 (3)	C20—C19—H19	121.6
C1—N1—C12	124.3 (3)	C18—C19—H19	121.6
C7—N2—C2	104.9 (3)	C19—C20—C21	123.7 (3)
C23—N3—C17	106.4 (2)	C19—C20—Br2	118.7 (3)
C23—N3—C28	129.2 (3)	C21—C20—Br2	117.6 (2)
C17—N3—C28	124.2 (3)	C22—C21—C20	120.0 (3)
C23—N4—C18	105.2 (3)	C22—C21—H21	120.0
N1—C1—C6	131.8 (3)	C20—C21—H21	120.0
N1—C1—C2	105.7 (3)	C21—C22—C17	116.7 (3)
C6—C1—C2	122.5 (3)	C21—C22—H22	121.6
C3—C2—N2	129.9 (3)	C17—C22—H22	121.6
C3—C2—C1	120.0 (3)	N4—C23—N3	112.8 (3)
N2—C2—C1	110.1 (3)	N4—C23—C240	118 (2)
C4—C3—C2	116.6 (3)	N3—C23—C240	129 (2)
C4—C3—H3	121.7	N4—C23—C24	123.2 (3)
C2—C3—H3	121.7	N3—C23—C24	124.0 (3)
C3—C4—C5	123.9 (3)	C240—C23—C24	6 (3)
C3—C4—Br1	118.0 (2)	N3—C28—C29	114.4 (3)
C5—C4—Br1	118.1 (2)	N3—C28—H28A	108.7
C6—C5—C4	119.8 (3)	C29—C28—H28A	108.7
C6—C5—H5	120.1	N3—C28—H28B	108.7
C4—C5—H5	120.1	C29—C28—H28B	108.7
C5—C6—C1	117.2 (3)	H28A—C28—H28B	107.6

### Hydrogen-bond geometry (Å, º)

Cg2 and Cg4 are the centroids of the S2,C13–C16 and S4,C29–C32 rings, respectively.

**Table:**

*D*—H···*A*	*D*—H	H···*A*	*D*···*A*	*D*—H···*A*
C12^i^—H12*B*^i^···N4	0.99	2.57	3.454 (4)	149
C22—H22···N2	0.95	2.61	3.504 (4)	158
C28—H28*A*···N2	0.99	2.61	3.522 (4)	152
C6^i^—H6^i^···N4	0.95	2.65	3.547 (4)	157
C3—H3···*Cg*4	0.95	2.68	3.578 (4)	158
C19—H19···*Cg*2^i^	0.95	2.62	3.512 (4)	157

Symmetry code: (i) *x*−1, *y*, *z*.
